# Metastatic Colorectal Cancer 2.0

**DOI:** 10.3390/cancers16122190

**Published:** 2024-06-11

**Authors:** Alessandro Passardi, Giorgia Marisi, Paola Ulivi

**Affiliations:** 1 Department of Medical Oncology, IRCCS Istituto Romagnolo per lo Studio dei Tumori (IRST) “Dino Amadori”, Via P. Maroncelli 40, 47014 Meldola, Italy; alessandro.passardi@irst.emr.it; 2 Biosciences Laboratory, IRCCS Istituto Romagnolo per lo Studio dei Tumori (IRST) “Dino Amadori”, Via P. Maroncelli 40, 47014 Meldola, Italy; paola.ulivi@irst.emr.it

Colorectal cancer (CRC) ranks third in frequency among cancers diagnosed in males and second in females [1]. Translational research has yielded notable advancements in treating patients with metastatic disease, with precision medicine emerging as a focal point in scientific inquiry. In particular, the integration of molecularly targeted therapies and antiangiogenic agents and the adoption of immune checkpoint inhibitors (ICIs) in the subgroup of patients with microsatellite instability have notably enhanced patient outcomes. Liquid biopsy presents a novel avenue for biomarker identification, potentially supplanting traditional tumor tissue analysis in clinical settings. Its utility extends to monitoring tumor burden and minimal residual disease, detecting tumor heterogeneity, and identifying molecular resistance to therapy [2]. The review by Gonzalez NS et al. (contribution 1) delves into the intricate landscape of CRC, exploring its heterogeneity, clonal evolution, and associated clinical ramifications. The authors elucidate the multifaceted nature of CRC, highlighting its diverse molecular subtypes, tumor evolution dynamics, and implications for patient management. Understanding CRC’s complexity is crucial in tailoring personalized therapeutic strategies, predicting treatment response, and mitigating disease progression. A holistic approach integrating genomic profiling, molecular characterization, and clinical parameters is needed to enhance patient outcomes.

Despite extensive translational research efforts spanning numerous years, predictive factors of efficacy for antiangiogenic agents in metastatic CRC (mCRC) remain elusive [3]. This persistent gap underscores the complexity of mCRC biology and highlights the need for continued investigation into novel biomarkers and predictive and prognostic models to guide treatment decisions and improve patient outcomes. In this context, the study by Moisuc DC et al. (contribution 2) investigates the prognostic significance of Cyclophilin A (CypA) in patients with mCRC undergoing treatment with bevacizumab and chemotherapy. Through a retrospective analysis of patient data, the research identifies CypA as an independent prognostic factor for survival in this patient population.

Approximately 5% of mCRC cases exhibit high MSI, resulting from deficient DNA mismatch repair, a characteristic associated with heightened sensitivity to immunotherapy, particularly ICIs [4]. However, the majority of mCRC cases are proficient in mismatch repair and exhibit microsatellite stability (MSS), a condition typically associated with resistance to ICIs. The review by Matteucci L. et al. (contribution 3) aims to offer a comprehensive overview of strategies aimed at overcoming resistance to ICIs and/or identifying subgroups of patients with MSS or dMMR mCRC who may derive benefit from immunotherapy.

The rising incidence of early-onset mCRC underscores the critical importance of understanding and effectively managing this challenging condition. The review by Medici B et al. (contribution 4) provides interesting insights into the epidemiology, clinical manifestations, and therapeutic approaches for early-onset mCRC. Improved diagnostic strategies and tailored treatment modalities are needed to optimize outcomes. The paper published by Storandt MH et al. (contribution 5) investigates the completion rates of genetic testing and the incidence of pathogenic germline mutations in patients with early-onset CRC through a single-institution analysis. In particular, the study sheds light on the prevalence of hereditary factors contributing to the disease. A comprehensive genetic evaluation is important in guiding personalized management strategies for these patients and might enhance screening and preventive measures in high-risk individuals.

Surgery plays a pivotal role in the management of mCRC, encompassing both the primary tumor and metastatic lesions. The primary tumor resection not only alleviates symptoms but also reduces the risk of complications such as obstruction or bleeding. Additionally, it’s a crucial component of multimodal treatment strategies, facilitating the delivery of systemic therapies and potentially improving outcomes [5]. The paper by Fanotto et al. (contribution 6) presents a pooled analysis of the TRIBE and TRIBE2 trials, examining the role of primary tumor resection in synchronous mCRC patients receiving upfront chemotherapy with bevacizumab. A notable association between primary tumor resection at baseline and favorable prognosis, along with a reduced occurrence of severe gastrointestinal and surgical adverse events during upfront chemotherapy combined with bevacizumab, emerges. Furthermore, the efficacy and safety profile of FOLFOXIRI plus bevacizumab remained consistent regardless of primary tumor resection status. Given the dearth of compelling evidence from randomized trials and the setbacks encountered in previous investigations, these findings advocate for the consideration of primary tumor resection in carefully selected asymptomatic patients.

In the context of metastatic disease, surgical resection of metastases, particularly in selected patients with limited metastatic burden and favorable prognostic factors, offers the opportunity for long-term survival and even cure. Furthermore, advances in surgical techniques, perioperative care, and multimodal treatment strategies, including neoadjuvant and adjuvant therapies, have expanded the indications for surgical intervention in mCRC. Despite these advancements, the optimal timing and extent of surgery in the metastatic setting remain subjects of ongoing research and debate, underscoring the need for multidisciplinary collaboration and individualized treatment approaches. The study by Canseco et al. (contribution 7) endeavors to furnish evidence supporting the notion that aggressive local interventions such as hepatic resection and/or radiofrequency ablation (RFA) confer substantial survival advantages for patients with CRC liver metastases (CRLM). Among the 2612 patients included in the analysis, 637 underwent hepatectomy, 93 underwent RFA, 92 underwent combined hepatectomy and RFA, and 1790 received non-aggressive treatment. Through Kaplan–Meier curves, multivariate Cox’s regression analysis, and frequency matching analysis, the study suggests that aggressive local control strategies in CRLM patients yield notable survival benefits, complementing systemic therapy. In this context, concerns have arisen regarding the optimal surgical approach for disappearing liver metastases (DLM) and small residual lesions encountered during chemotherapy. The study by Boraschi et al. (contribution 8) sought to assess the clinical outcomes of DLM and small remnant liver metastases (≤10 mm) identified through hepatobiliary contrast-enhanced and diffusion-weighted MR imaging (DW-MRI) in mCRC patients undergoing first-line chemotherapy. Results indicate that DLM likely signify a complete response in patients devoid of chemotherapy-induced sinusoidal obstruction syndrome. In such cases, follow-up with liver MRI is advisable, with resection recommended upon disease relapse. Surgical excision of small residual liver metastases should be pursued whenever technically feasible, emphasizing the importance of proactive intervention in managing these lesions.

In this Special Issue, several crucial aspects of mCRC research are addressed and discussed. An enhanced molecular understanding of CRC, based on genetic, epigenetic, and proteomic features, could enable the development of novel therapeutic strategies for this disease in the future. The integration of biological parameters with clinical and radiological factors through artificial intelligence tools will provide a more comprehensive and complete understanding of CRC, which could be beneficial to clinicians in improving patient management.
